# Practical Notes on Diagnosis and Treatment

**Published:** 1912-06-15

**Authors:** 


### PRACTICAL NOTES ON DIAGNOSIS AND TREATMENT.

**Atonic Dyspepsia.**

The following is a useful formula given about an hour after each of the principal meals:—Acid. Hydrochlor. dil. ♏xv, Acid. Carbolic grs. ij, Liq. Strychnin. Hydrochlor., ♏v, Tinct. Zingiberis ♏xv, Glycerin, ♏xx, Aq. Chloroform. ad ℥i.

**Vaginitis in Infants.**

This is difficult to treat in many cases. A weak solution of silver nitrate, protargol or argyrol is suitable to employ; and small bougies of ichthyol may be used.

**Leukoplakia**

This is by no means always due to syphilis. It means local irritation generally from tobacco. But smokers who have had syphilis are more likely to develop it than the non-syphilitic.

**Leeches in Iritis.**

In iritis and other inflammatory affections of the eye, said the late Dr. Bell Taylor, it is difficult nowadays to persuade patients to submit to venesection, but "I have applied 70 leeches to the temple in one case and have in many cases taken many ounces of blood with Herteloup's instrument from the same situation and with the utmost benefit."—*Lancet.*

**Chronic Colitis.**

In this condition a preparation known as Honthin is sometimes useful as it exercises an astringent action on the mucous membrane of the intestine. It is a brown insoluble powder consisting of a combination of tannin and keratin. The dose is 20 grains thrice daily. As it is tasteless, the powder may be placed on the tongue and followed by a drink of milk or water.

**Hæmoptysis.**

If this is severe amyl nitrite ought to be inhaled. By dilating the systemic arteries it lowers the pressure in the pulmonic circuit. Rest, of course, is essential and very often morphine.

**Leucocytosis in Pneumonia.**

It is not uncommon to have difficulty in interpreting a condition of pyrexia unaccompanied by physical signs; and one of the possibilities of such a position is pneumonia. A count of the leucocytes is sometimes helpful, as a definite leucocytosis is usual in pneumonia, whilst this is not the case either in typhoid fever or influenza. The leucocytosis is due to an increase of the polymorphs and rises daily until about the time of the crisis.

**Gonorrhœal Arthritis.**

This may exhibit itself as a polyarthritis very like an attack of sub-acute rheumatism, but the pain does not subside in one set of joints as it appears in another set, and sodium salicylate does not afford substantial relief. In other cases the arthritis is limited to a single joint; such a condition should always provoke suspicion, even if the patient is a child. Sometimes arthritis appears in cases of ophthalmia neonatorum; the prognosis here is good provided the conjunctival condition is effectively treated.

**To Estimate the Size of the Stomach.**

The readiest fashion to effect this is to give 120 grains of sodium bicarbonate in solution followed by 90 grains of tartaric acid in solution; less quantities are not sufficient. It is necessary that the stomach should be empty when the test is applied or otherwise vomiting may be produced.

**Prognosis in Pleural Effusion.**

The statement, often made, that most cases of pleurisy with serous effusion are of tubercular origin is perhaps correct. But it is apt unduly to weight the prognosis. Provided there is no history of tuberculosis the outlook in such cases is distinctly good; even if tubercle is the basis it is usually of a mild type, and the patient makes a good recovery. This is the case even when the febrile process extends over some weeks.

**Chloretone.**

There can be no doubt that this drug is valuable in chorea. Five grain doses in half an ounce of petroleum emulsion every four or six hours will quieten the movements in the course of three or four days; the dose should then be reduced. Chloretone is also of value to prevent sea-sickness.

**Retention of Urine.**

Mr. Harford Edwards has found that a rectal injection of glycerine is usually successful in cases of retention of urine in hysteria and after labour.—*British Medical Journal.*

**Chloral Hydrate.**

When taken continuously chloral tends to lessen the alkalinity of the blood as it yields a certain amount of formic acid. Hence potassium or sodium bicarbonate should always be prescribed when chloral is used regularly.

**Pruritus.**

A 20 per cent. solution of bromocoll is recommended in cases of nervous pruritus and in other conditions giving rise to itching. For pruritus ani, as well as the solution, a suppository containing bromocoll 10 grains, cacao butter 20 grains, may be ordered.

**Thrombosis in Typhoid Fever.**

This, Sir Almroth Wright has suggested, largely depends on an excessive amount of calcium in the blood, which, in turn, arises from the large amount of milk administered as food. To prevent such thrombosis, he recommends the partial decalcification of the milk by the addition of 20 to 40 grains of sodium citrate to each pint; this also renders the milk more easily digested.

